# Autophagy: A novel therapeutic target for hepatocarcinoma (Review)

**DOI:** 10.3892/ol.2014.1916

**Published:** 2014-02-26

**Authors:** ZHANGGUI WANG, WEIDONG HAN, XINBING SUI, YONG FANG, HONGMING PAN

**Affiliations:** Department of Medical Oncology, Sir Run Run Shaw Hospital, Zhejiang University, Hangzhou, Zhejiang 310016, P.R. China

**Keywords:** autophagy, hepatocarcinoma, anti-hepatocarcinoma therapy

## Abstract

Autophagy is a highly conserved intracellular degradation process and plays an important role in hepatocarcinogenesis. Available data show that autophagy is involved in anti-hepatocarcinoma (HCC) therapies. Autophagy regulation involves a novel target for overcoming therapeutic resistance and sensitizing HCC to currently therapeutic methods. This is a systematic review on the interface of autophagy and the development of HCC and outlining the role of autophagy in current anti-HCC approaches. Understanding the significance of autophagy in anti-HCC therapy may offer a novel therapeutic target for improving anti-cancer efficacy and prolong survival for HCC patients.

## 1. Introduction

Autophagy is a conserved intracellular degradation process in which cellular organelles, proteins and invading microbes are degraded by lysosomes. According to the routines of target cargos delivered to lysosome, there are three types of autophagy: macroautophagy, mitoautophagy and chaperone-mediated autophagy (1). This review focused on macroautophagy, hereafter referred to as autophagy.

Autophagy is a multifaceted process, consisting of sequential stages, including initiation, elongation, maturation and degradation, which are regulated by a series of highly conserved autophagy-related genes (Atgs) involved in various signaling pathways (2–4). Autophagy is characterized by the formation of double-membrane vesicles, known as autophagosomes, which are engulfed by cytoplasmic molecules. Subsequently, the autophagosome fuses with the lysosome, which provides hydrolases and the sequestered contents undergo degradation and recycling. Autophagy contributes to the pathogenesis of diverse diseases, such as neuronal degeneration, inflammatory bowel disease, aging and cancer (5–8). Autophagy is involved in tumor development and progression, however, its exact role remains to be elucidated. Based on current information, autophagy plays a dual role in cancer initiation and development. First, autophagy eliminates senescent and injured cells, thereby limiting chromosomal instability and suppresses tumor initiation. Deletion of Atgs in mice results in a high incidence of spontaneous tumors (9). Second, autophagy could provide energy by recycling damaged organelles, DNA, aggregated proteins and pathogens to maintain energy balance, which promotes cancer cell survival. As a result, the inhibition of autophagy may be a novel strategy to improve the efficacy of anti-cancer therapy.

Primary liver cancer is the fifth most common cancer worldwide and the third most common cause of cancer-related mortality (10). Hepatocarcinoma (HCC) is the most common primary malignancy of hepatocytes which accounts for ~90% of primary liver cancers (11). Most cases of HCC (~80%) are associated with chronic hepatitis B virus (HBV) or hepatitis C virus (HCV) infection (11–13). In addition, non-alcoholic and alcoholic fatty liver disease contribute to the development of HCC (14). Surgical resection or liver transplantation remains the mainstay of treatment for early HCC patients. However, the majority of patients present at an advanced stage, and only a few newly diagnosed HCC patients are eligible for chemotherapy, targeted therapy, transcatheter arterial chemoembolization (TACE) or radiofrequency ablation.

Autophagy in HCC has been previously investigated. Dysregulation of autophagy is involved in hepatitis, fibrosis, cirrhosis and HCC (15–17). Modulation of autophagy can affect the efficacy of anti-HCC therapy. Therefore, it is crucial to understand the potential mechanisms underlying the involvement of autophagy in the development, progression and anti-cancer therapy of HCC, which may lead to novel therapeutic approaches for liver cancer. This review aimed to provide an overview of current available information regarding the role of autophagy in the development of HCC and the effect of autophagy in anti-HCC therapy.

## 2. The role of autophagy in HCC

Since autophagy is a stress response, it is associated with the development of HCC (16). Thus, understanding the role and potential molecular mechanisms underlying the involvement of autophagy in HCC formation and development, which may provide novel therapeutic strategies for HCC, is crucial.

### Autophagy is involved in the formation of HCC

The formation of HCC is a multi-stage process, which frequently develops in patients suffering from chronic liver injury caused by chronic alcohol consumption and hepatitis B or C infections (18). These conditions result in the death of healthy liver cells and the initiation of an inflammatory response that sequentially induces liver cell proliferation, subsequently compensating cirrhosis and eventually the development of HCC.

Recent studies have demonstrated that almost all factors leading to chronic liver injury or inflammation were capable of inducing autophagy. Autophagy is involved in hepatic lipid and alcohol metabolism (19,20). In Atg7-specific knockdown mice, lipid was markedly deposited in hepatocytes (19). Ding *et al* (21) found that acute ethanol administration promoted the removal of lipid droplets and damaged mitochondria by the induction of autophagy in mouse hepatocytes. Suppression of autophagy exacerbated alcoholic liver injury.

Epidemiological, clinical and experimental studies have demonstrated that the relative risk of HCC in HBsAg carriers is >200 times that in matched non-carriers (22,23). HBV can enhance autophagy in Huh7 and HepG2 cells in mouse orthotopic liver cancer models (24,25). The HBV X protein sensitizes hepatoma cells to starvation-induced autophagy via the upregulation of Beclin-1 expression (24,26). In addition, HBV promotes viral replication by the binding of HBx and PI3KC3 (26). Recent findings suggest that autophagy is involved in HCV infection (27–29). Inhibition of autophagy abrogates the replication of HCV by siRNA-targeting Atgs (30). HCV induces the accumulation of autophagosome in hepatoma cells by unfolded protein response (UPR) (27).

Liver fibrosis is the final result of liver injury or chronic liver disease, which ultimately progresses to liver cirrhosis and cancer. Induction of autophagy promotes hepatic stellate cell (HSC) proliferation or activation, which is transited to myofibroblast when it is activated under the conditions of liver hepatitis, alcohol or non-alcohol liver diseases (31). Pharmacological inhibitors baflomycin A1, 3-methyladenine (3-MA) or chloroquine (CQ) suppress the activation and proliferation of HSC *in vitro* and *in vivo*.

**Figure 1:**
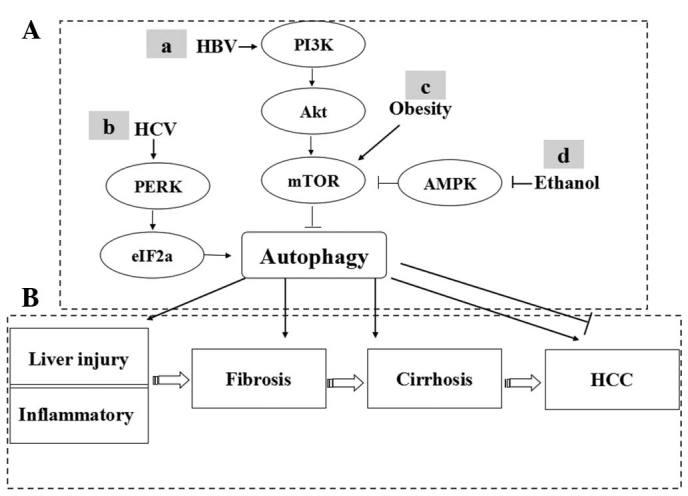
Autophagy is involved in the initiation of hepatocarcinoma (HCC). (A) Autophagy involvement in etiological factors of HCC. (a) Autophagy is induced by hepatitis B virus (HBV). Activating PI3K signal is one of the mechanisms in autophagy induced by HBV (25,26). (b) Autophagy is activated in hepatocytes infected with hepatitis C virus (HCV) through the activation of unfolded protein response signaling (27). (c) Autophagy is suppressed in obesity in hepatocytes possibly because the mTOR signaling pathway is overactivated by the metabolism of overnutrition (20). (d) Autophagy is inhibited by ethanol, which may be caused through the downregulation of AMPK activity (21). (B) A multi-stage process in the formation of HCC.

Collectively, autophagy is involved in chronic liver disease caused by non-alcoholic and alcohol factors, as well as HBV or HCV infection. Various potential signaling pathways are involved (Fig. 1).

### Autophagy plays a dual role in hepatocarcinogenesis

Despite the literature available, the role of autophagy in hepatocarcinogenesis remains controversial. Since autophagy is a stress response and survival mechanism, mouting evidence demonstrates that autophagy contributes to the survival of cancer (8,32). It has been reported that autophagy was increased in tumor interior rather than in cancer margins, contributing to the survival of interior tumor cells under an hypoxic-ischemic environment (32). Microtubule-associated protein light chain 3 (LC3) was significantly highly expressed in HCC compared with non-cancerous tissues, and was also significantly correlated with tumor size. In addition, LC3 was an independent predictor of HCC recurrence after surgery only in the context of large tumors (33). Similarly, increased levels of LC3-II were observed in HCC tissues with low glucose uptake and a high K-Ras expression (34). Collectively, these data support the hypothesis that autophagy serves to maintain tumor survival.

As an essential regulator for cellular homeostasis, autophagy plays an important role in carcinogenesis. It has been well-documented that autophagy is a tumor suppressor that acts as a housekeeping gene (35). Mice with homozygous Beclin-1 knockout have a high incidence of spontaneous tumors, such as HCC (36). Similarly, the deletion of Atg5 or Atg7 in liver, two key elements of autophagy elongation, resulted in the increasing incidence of HCC (37). The expression and activity of Atg5 or Atg7 are reduced in HCC cell lines compared with normal hepatocytes *in vitro* (38). Kotsafti *et al* (37) found that the decreased expression of Beclin-1 was observed in human HCC tissue and was correlated with recurrent disease and free-disease survival (37). These findings establish a role for autophagy as a suppressor in HCC.

**Figure 2:**
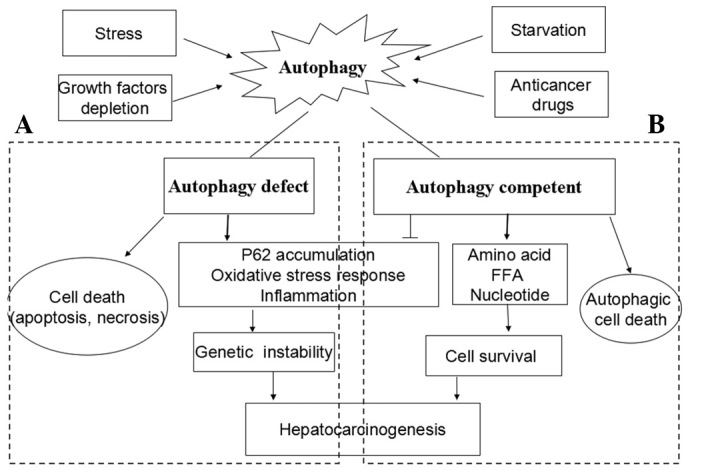
The dual role of autophagy in the development of hepatocarcinoma (HCC). Autophagy is activated as a response to stress, growth factors depletion, starvation and anti-tumor treatment. (A) Under autophagy-deficient conditions, cells succumb to death when challenged with death stimuli. Thus, autophagy acts as a tumor suppressor. On the other hand, proteins scavenged by autophagy accumulate and result in genetic instability, which in turn promote hepatocarcinogenesis. (B) Under autophagy-competent conditions, cells succumb to survival when challenged with death stimuli. Autophagy removes damaged organelles, misfolded and aggregated proteins, both of which generate free fatty acids and amino acids that can provide energy to facilitate hepatocarcinogenesis. However, the sustained activation of autophagy leads to autophagic cell death, termed as type II programmed cell death. FFA, free fatty acids.

Autophagy is known to suppress tumorigenesis in healthy cells, albeit it contributes to the survival of an established tumor (Fig. 2).

## 3. Autophagy and anti-HCC therapy

Due to the controversial role it plays in the initiation and development of HCC, autophagy has become an emerging and noteworthy field of study for identifying treatment for HCC. Appreciation of the function of autophagy in cancer treatment is critical, because anticancer therapies have been shown to initiate autophagy *in vitro* and *in vivo*.

### Autophagy in chemotherapy

Currently, chemotherapy is almost ineffective for HCC because of the inherent or acquired chemoresistance and limitation of liver function. Autophagy is known to promote cancer resistance to chemotherapy. Guo *et al* (39) reported that cisplatin or 5-FU induced autophagy in HepG2, SMMC-7721 and Hep3B cells. Autophagy inhibition by 3-MA or the siRNA targeting Beclin-1 increased chemotherapy-induced apoptosis by causing significant damage of mitochondrial membrane *in vitro* and *in vivo*. Oxaliplatin-based combination chemotherapy has shown promising anti-tumor activities in patients with HCC (40). Ding *et al* (41) found that autophagy was activated by oxaliplatin in the HCC cells. Suppression of autophagy with pharmacologic inhibitors or siRNAs targeting essential autophagic genes enhanced cell death induced by oxaliplatin in HCC cells, which correlated with the generation of reactive oxygen species.

However, adriamycin, which is routinely used as a monotherapy for advanced HCC, induced autophagic cell death rather than cytoprotective autophagy in Hep3B cells (42). It is known that the MAPK/ERK pathway, which is upregulated in HCC, can regulate autophagy (43). The potential mechanism of autophagic cell death induced by adriamycin is present in the sustained activation of the MAPK/ERK pathway, which leads to autophagic progression, followed by an irreversible stage and ultimately cell death.

Autophagy is known to serve as a protective mechanism under chemotherapeutics (39–41). Although autophagic cell death has been reported, this should be defined carefully in its particular context and the results should be elucidated prudently.

### Autophagy in molecular-targeted therapy

Molecular-targeted therapy is critical for advanced or recurrent HCC. Sorafenib, a multi-targeted receptor tyrosine kinase inhibitor (TKI) that targets Ras, VEGFR and PDGFR, was approved as the standard therapy for advanced unresectable HCC (44). However, sorafenib only provides modest effects, prolonging survival in patients with HCC from a median of 7.9 to 10.7 months (45,46). Sorafenib induced the accumulation of autophagosomes in HCC cells through inhibition of the mTORC1 pathway (47,48). The underlying molecular mechanisms of this process are: i) The PI3K/Akt/mTOR signaling pathway is capable of regulating autophagy. Besides the Raf/MEK/MAPK pathway, sorafenib inhibits activation of the mTORC1 pathway, which ultimately stimulates a series of signals to induce autophagy. ii) The endoplasmic reticulum (ER) is an essential intracellular organelle required for the synthesis and quality control of proteins. Findings of recent studies have demonstrated that autophagosome membranes originate from ER, thereby suggesting a direct connection between the ER and autophagy (49). Shi *et al* (48) reported that sorafenib significantly increased the mRNA and protein expression levels of the UPR target genes IRE-1 and CHOP as well as eIF2α phosphorylation. Thus, sorafenib-triggered ER stress is critical for autophagy activation. Briefly, autophagy conferred a survival advantage for sorafenib treatment in HCC, which may be an attractive strategy for HCC treatment. Similarly, autophagy exerts a cytoprotective effect in HCC cell lines treated with proteasome inhibitor carbobenzoxy-Leu-Leu-leucinal (MG-132), bortezomib, or bevacizumab, a humanized monoclonal antibody that binds VEGF-A (50–52).

It has, however, been demonstrated that autophagic cell death was a major contributor to molecular-targeted therapies associated with the anti-proliferative effect on tumor cells. Tai *et al* (53) found that sorafenib and SC-59, a novel sorafenib derivative, disrupt myeloid cell leukemia-1 (Mcl-1) associated with Beclin-1 and promote significant autophagic cell death. OSU-03012, a highly selective COX-2 inhibitor, induced reactive oxygen species-related autophagy to inhibit HCC cell proliferation (54). Nilotinib, a second-generation TKI for leukemia, induced autophagic cell death in HCC by deactivating phosphatase PP2A and increasing AMPK phosphorylation. Autophagy inhibition by hydroxychloroquine (HCQ) reduced the effect of nilotinib *in vivo* (55).

Collectively, molecular-targeted therapy activates autophagy in HCC cells and autophagy can function to promote either tumor cell survival or cell death.

### Autophagy in radiotherapy

Conformal radiotherapy (RT) and stereotactic body radiation therapy are used to treat single or solitary liver metastases or unresectable HCC in some preferential patients. A phase II trial demonstrated that 48% of patients who had HCC or local metastases, unsuitable for or refractory to standard therapy and received palliative RT, exhibited improvement in symptoms such as pain, abdominal discomfort, nausea, or fatigue (56). In recent studies, it was shown that genetic or pharmacological interference with autophagy can enhance the response to radiation in renal cell carcinoma, breast cancer, head and neck squamous cell carcinoma and glioblastoma (57–59). Iron radiation contributed to a cytoprotective autophagy that could be inhibited by CQ or by the silencing of autophagy-regulatory genes, with the consequent enhancement of radiation sensitivity in breast cancer (59,60). By contrast, Altmeyer *et al* (61) found that irradiation with fast neutrons, which are high-linear energy transfer (LET) particles, induced autophagic cell death in the human HCC SK-Hep1 cells (61). Furthermore, autophagy plays a pivotal role in cell death after high-LET irradiation in orthotopic human hepatocellular carcinoma (62). Briefly, autophagy can be induced by radiation therapy, which functions to protect or promote cell death. However, the potential mechanism underlying this role remains to be determined.

### Autophagy in TACE or photodynamic therapy

TACE is used in unresectable HCC, as well as pre- or post-operative adjuvant therapy in patients with resectable HCC to improve survival. Studies have shown that LC3 expression was significantly higher after TACE compared to tumors that had not undergone treatment in human HCC tissue samples (41). Autophagy inhibitor CQ combined with TACE represented better outcomes compared to TACE alone in a rabbit VX2 liver tumor model (63).

Photodynamic therapy (PDT) is a process whereby the interaction between photodynamic agents localized in neoplastic tissues and oxygens in tissues was initiated by irradiation at appropriate wavelength (64). Using a murine hepatoma 1c1c7 model, Andrzejak *et al* (65) found PDT-induced autophagy was cytoprotective since PDT efficacy was significantly enhanced in Atg7-knockdown cells.

### Autophagy in immunotherapy

During the process of tumor development, tumor antigens are not visible to T cells thereby escaping immune surveillance (66). Thus, immunotherapy is considered a promising therapeutic option with the aim of inducing or increasing HCC-specific immune response and overcoming immune escape, demonstrating the importance of autophagy in central aspects of the immune response, making it an attractive target for cancer therapy. Cytokines such as IFN-γ, IL-12 and TNF-β, are important effector components in the immune response (67). IFN-γ, which plays an important role in HCC immunotherapy, inhibited liver cancer cell growth by the induction of autophagic cell death. Knockdown of the Beclin-1 or Atg5 attenuated the inhibitory effect of IFN-γ (68). IL-2, a major regulator of immunotherapy that was approved for advanced renal cancer and melanoma, can increase autophagy flux in murine liver (67). The combination of IL-2 with CQ prolonged survival in a murine metastatic liver tumor model. The potential mechanism involved is that CQ inhibited oxidative phosphorylation and ATP production and promoted apoptosis of cancer cells (67). Li *et al* (68) reported that toll-like receptor-2 (TLR-2) deletion sensitized liver cancer cells to diethylnitrosamine, a genotoxic carcinogen that can induce HCC. TLR-2 deficiency caused a decrease in the expression of IFN-γ, IL-6 as well as suppression of the autophagic flux. Restoring autophagic flux by treating TLR2-deficient mice with IFN-γ, a T-helper 1 (Th1) cytokine and positive modulator of autophagy, attenuated the carcinogenesis and progression of HCC in TLR2-deficient mice (68). Recently, a cancer vaccine originating from tumor cell-derived autophagosomes (DRibbles) combined with dendritic cells (DCs) has shown a specific T-cell response against HCC and resulted in the significant inhibition of tumor growth compared to mice treated with DCs alone (69).

### Autophagy in liver transplantation

Liver transplantation is a widely accepted treatment for HCC patients and is the best available option for early HCC. Ischemia/reperfusion (I/R) injury occurs during the procedure of liver transplantation, which is the main cause of initial deficiencies and primary dysfunction of liver grafts (70). Accumulating evidence suggests that CQ administration worsens I/R injury via autophagy inhibition in kidney and heart after ischemia (71,72). It was also demonstrated that CQ treatment ameliorated liver I/R injury in the early phase of reperfusion but worsened liver I/R injury in the late phase via inhibition of autophagy on rat hepatic I/R injury (32). Hepatocytes that possessed abundant autophagosomes often underwent autophagic cell death which triggered liver graft dysfunction (73).

By contrast, Degli Esposti *et al* (74) demonstrated that ischemic preconditioning induces autophagy in human steatotic liver grafts and reduces rejection in recipients. Rapamycin, a key immunosuppressive drug and autophagy inducer, improved the survival of HCC patients with liver transplantation (75).

Thus, whether autophagy functions in cell survival or death in anti-HCC therapy is highly dependent on the cell type, mechanisms of agents and the signaling pathways (Table I).

## 4. Autophagy modulation and anti-HCC therapy

Although the role of autophagy in hepatocarcinogenesis and treatment thereof has been outlined in detail, autophagy modulation based on its function may provide novel opportunities for HCC treatment. Autophagy inhibition is an emerging strategy that enhances cytotoxicity in combination with anti-HCC therapies in the prosurvival function of autophagy. By contrast, the activation of autophagy is another method to facilitate the anti-tumor effect combination with current therapeutic methods in autophagic cell death.

### Inhibiting autophagy in anti-HCC therapy

Recent studies have reported that genetic or pharmacological interference with autophagy can enhance the response to chemotherapy, molecular-targeted and radiation therapy (50,52,59). CQ and HCQ, which are used in malaria, are commonly used as autophagy inhibitors in various tumor experiment models (76). In pre-clinical and clinical trials conducted, the role of autophagy inhibition through pharmacologic inhibitors such as CQ and HCQ was examined in various tumors (www.clinicaltrials.gov). As mentioned above, autophagy inhibitors can enhance the effectiveness of oxaliplatin, cisplatin, 5-FU and sorafenib in HCC models (39,41,47). The coadministration of oxaliplatin and CQ induced a marked decrease in tumor volume compared with either agent alone in HCC xenografts (41). CQ interacted synergistically with bortezomib to suppress tumor growth to a greater extent in HCC experimental models (51). The concomitant inhibition of autophagy by CQ or genetic knockdown Atg7 sensitized hepatoma cells to sorafenib (47). Similarly, autophagy suppression by means of 3-MA and inactive Atg4B inhibited proliferation in Huh7 cells (77). Thus, autophagy inhibition is an attractive strategy for overcoming therapeutic resistance in the protective functions of autophagy.

### Inducing autophagic cell death in anti-HCC therapy

Sustained activation of autophagy may kill cancer cells with a high apoptotic defect, termed autophagic cell death (78). Autophagic cell death has been observed in malignant glioma cells treated with arsenic trioxide or sodium selenite (79,80). Vorinostat, a histone deacetylase inhibitor, induced autophagic cell death in the U937 hematological cell line (81). Autophagy activation may serve as an alternative strategy for eliminating cancer cells, especially HCC cells with apoptotic defect. As discussed above, sorafenib induced autophagic cell death through the Mcl-1 signaling pathway (53). Under context-specific conditions, the sustained upregulation of autophagy may benefit from sorafenib treatment. However, evidence from *in vivo* studies and clinical trials are relatively limited and whether the induction of autophagic cell death in tumor cell death can be sensitized to HCC therapy remains unclear.

**Figure 3:**
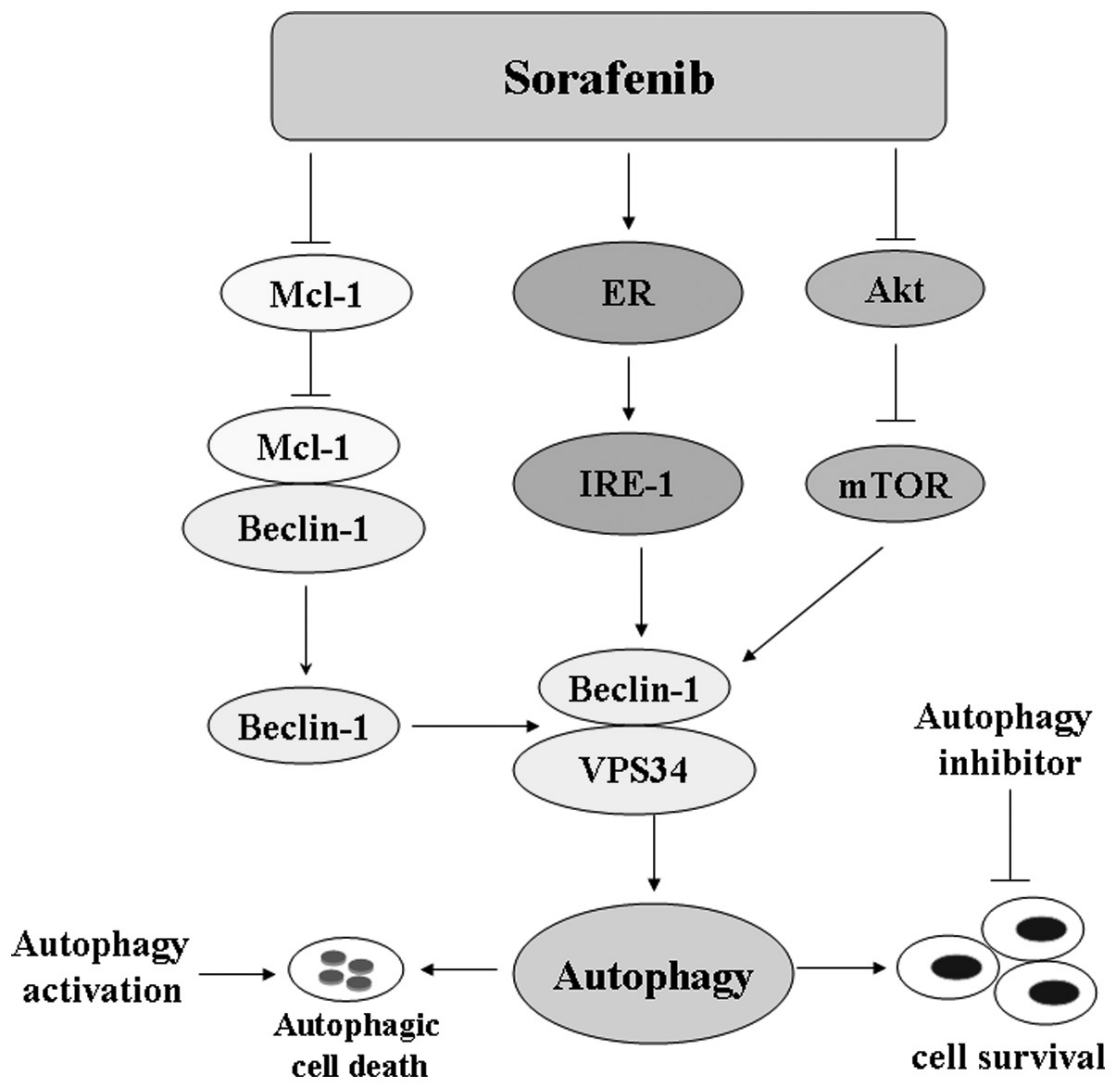
Sorafenib activates autophagy through different signaling pathways. Autophagic cell death is triggered by sorafenib by suppressing myeloid cell leukemia-1 (Mcl-1)-related signaling pathway in hepatocarcinoma (HCC) PLC5, SK-Hep1, HepG2 and Hep3B cells (53). Thus, inducers of autophagy may be used in combination with sorafenib to promote anti-cancer efficacy. Cytoprotective autophagy is triggered by sorafenib through endoplasmic reticulum (ER) or the Akt/mTOR signaling pathway in MHCC97-L and PLC/PRF/5 cells (47,48). Inhibitors of autophagy may therefore be used in combination with sorafenib to promote anti-cancer efficacy.

Taken together, although connections between autophagy and anti-HCC therapies have been suggested, autophagy modulation provides new prospects in anti-HCC therapy. The complexity of autophagy in hepatocarcinogenesis and anti-HCC therapies, however, makes it difficult to define how to regulate autophagy (inhibition or activation) in order to ensure maximum therapeutic advantage. A typical example is that sorafenib-induced autophagy is particularly context-dependent and exhibits an opposite function through different signaling pathways (Fig. 3).

## 5. Conclusions

The role of autophagy in cancer remains controversial and highly context-dependent. As outlined in this review, autophagy plays a dual role in multiple aspects to the sequential process of liver cancer initiation, promotion, progression and metastasis. In addition to this, autophagy is induced through various types of anti-HCC therapies. Previous studies (34,37) have demonstrated that autophagy plays an anti-tumor effect in suppression of the formation of HCC, while serving as a pro-survival mechanism to promote liver cancer development, and results in resistance to anti-HCC therapy. Thus, targeting autophagy is a promising strategy for liver cancer therapy.

Limitations to the clinical application of autophagy in anti-HCC therapy should first be overcome. Although it is widely accepted that anti-tumor treatment induces autophagy, it remains to be determined whether this activation promotes cell survival as a response to stress, or leads to cell death under the condition of apoptotic defects. Therefore, obtaining the function status of autophagy in anti-HCC treatment may contribute to devising a rationale for the treatment of HCC. Additionally, whether autophagy modulation (inhibition or activation) increased the susceptibility to treatment in healthy cells or eradicated the balance of homeostasis should be clarified. As an autophagy inhibitor, CQ sensitizes the normal renal proximal tubular cells to cisplatin administration (82). Rapamycin, an autophagy inducer, is also an immunosuppressor (83). Selection of a suitable drug that targets autophagy in order to enhance the efficacy of anti-HCC therapy remains a challenge. Subsequently, coadministration of the autophagy regulator with anti-HCC therapy may also aid in the elucidation of the antistatic effect. Thus, targeting autophagy remains a promising interventional strategy for the treatment of HCC.

## Figures and Tables

**Table I:** The functional status of autophagy in hepatocarcinoma treated with different agents in experiments.

Agents	Autophagy	Function	(Refs.)
Chemotherapy			
Cisplatin	↑	Cell survival	(39)
5-FU	↑	Cell survival	(39)
Oxaliplatin	↑	Cell survival	(41)
Adriamycin	↑	Cell death	(42)
Targeting therapy			
Sorafenib	↑	Cell survival	(47,48)
	↑	Cell death	(53)
MG-132	↑	Cell survival	(50)
Bevacizumab	↑	Cell survival	(51)
Bortezomib	↑	Cell survival	(52)
OSU-03012	↑	Cell death	(54)
Nilotinib	↑	Cell death	(55)
Immunotherapy			
IFN-γ	↑	Cell death	(68)
IL-2	↑	Cell death	(67)
DRibble	↑	Cell survival	(69)

## References

[1] Degenhardt K, Mathew R, Beaudoin B (2006). Autophagy promotes tumor cell survival and restricts necrosis, inflammation, and tumorigenesis. Cancer Cell.

[2] Kroemer G, Marino G, Levine B (2010). Autophagy and the integrated stress response. Mol Cell.

[3] Rosenfeldt MT, Ryan KM (2011). The multiple roles of autophagy in cancer. Carcinogenesis.

[4] Mizushima N (2007). Autophagy: process and function. Genes Dev.

[5] Yue Z (2007). Regulation of neuronal autophagy in axon: implication of autophagy in axonal function and dysfunction/degeneration. Autophagy.

[6] Scharl M, Rogler G (2012). Inflammatory bowel disease: dysfunction of autophagy?. Dig Dis.

[7] Yamaguchi O, Otsu K (2012). Role of autophagy in aging. J Cardiovasc Pharmacol.

[8] Eskelinen EL (2011). The dual role of autophagy in cancer. Curr Opin Pharmacol.

[9] Liang C, Jung JU (2010). Autophagy genes as tumor suppressors. Curr Opin Cell Biol.

[10] Yang JD, Roberts LR (2010). Hepatocellular carcinoma: a global view. Nat Rev Gastroenterol Hepatol.

[11] Fares N, Peron JM (2013). Epidemiology, natural history, and risk factors of hepatocellular carcinoma. Rev Prat.

[12] Guerrieri F, Belloni L, Pediconi N (2013). Molecular mechanisms of HBV-associated hepatocarcinogenesis. Semin Liver Dis.

[13] Yamazaki K, Masugi Y, Sakamoto M (2011). Molecular pathogenesis of hepatocellular carcinoma: altering transforming growth factor-beta signaling in hepatocarcinogenesis. Dig Dis.

[14] Maillard E (2011). Epidemiology, natural history and pathogenesis of hepatocellular carcinoma. Cancer Radiother.

[15] Ni HM, Williams JA, Yang H (2012). Targeting autophagy for the treatment of liver diseases. Pharmacol Res.

[16] Cui J, Gong Z, Shen HM (2013). The role of autophagy in liver cancer: molecular mechanisms and potential therapeutic targets. Biochim Biophys Acta.

[17] Rautou PE, Mansouri A, Lebrec D (2010). Autophagy in liver diseases. J Hepatol.

[18] Cabibbo G, Maida M, Genco C (2012). Natural history of untreatable hepatocellular carcinoma: a retrospective cohort study. World J Hepatol.

[19] Singh R, Kaushik S, Wang Y (2009). Autophagy regulates lipid metabolism. Nature.

[20] Dolganiuc A, Thomes PG, Ding WX (2012). Autophagy in alcohol-induced liver diseases. Alcohol Clin Exp Res.

[21] Ding WX, Li M, Yin XM (2011). Selective taste of ethanol-induced autophagy for mitochondria and lipid droplets. Autophagy.

[22] Lavanchy D (2004). Hepatitis B virus epidemiology, disease burden, treatment, and current and emerging prevention and control measures. J Viral Hepat.

[23] Beasley RP (1988). Hepatitis B virus. The major etiology of hepatocellular carcinoma. Cancer.

[24] Sir D, Tian Y, Chen WL (2010). The early autophagic pathway is activated by hepatitis B virus and required for viral DNA replication. Proc Natl Acad Sci USA.

[25] Tian Y, Sir D, Kuo CF (2011). Autophagy required for hepatitis B virus replication in transgenic mice. J Virol.

[26] Tang H, Da L, Mao Y (2009). Hepatitis B virus X protein sensitizes cells to starvation-induced autophagy via up-regulation of beclin 1 expression. Hepatology.

[27] Shinohara Y, Imajo K, Yoneda M (2013). Unfolded protein response pathways regulate Hepatitis C virus replication via modulation of autophagy. Biochem Biophys Res Commun.

[28] Sir D, Kuo CF, Tian Y (2012). Replication of hepatitis C virus RNA on autophagosomal membranes. J Biol Chem.

[29] Shrivastava S, Bhanja Chowdhury J, Steele R (2012). Hepatitis C virus upregulates Beclin1 for induction of autophagy and activates mTOR signaling. J Virol.

[30] Dreux M, Gastaminza P, Wieland SF, Chisari FV (2009). The autophagy machinery is required to initiate hepatitis C virus replication. Proc Natl Acad Sci USA.

[31] Thoen LF, Guimaraes EL, Dolle L (2011). A role for autophagy during hepatic stellate cell activation. J Hepatol.

[32] Fang H, Liu A, Dahmen U, Dirsch O (2013). Dual role of chloroquine in liver ischemia reperfusion injury: reduction of liver damage in early phase, but aggravation in late phase. Cell Death Dis.

[33] Yang JD, Seol SY, Leem SH (2011). Genes associated with recurrence of hepatocellular carcinoma: integrated analysis by gene expression and methylation profiling. J Korean Med Sci.

[34] Kim JH, Kim HY, Lee YK (2011). Involvement of mitophagy in oncogenic K-Ras-induced transformation: overcoming a cellular energy deficit from glucose deficiency. Autophagy.

[35] Rosenfeldt MT, Ryan KM (2009). The role of autophagy in tumour development and cancer therapy. Expert Rev Mol Med.

[36] Qu X, Yu J, Bhagat G (2003). Promotion of tumorigenesis by heterozygous disruption of the beclin 1 autophagy gene. J Clin Invest.

[37] Kotsafti A, Farinati F, Cardin R (2012). Autophagy and apoptosis-related genes in chronic liver disease and hepatocellular carcinoma. BMC Gastroenterol.

[38] Takamura A, Komatsu M, Hara T (2011). Autophagy-deficient mice develop multiple liver tumors. Genes Dev.

[39] Guo XL, Li D, Hu F (2012). Targeting autophagy potentiates chemotherapy-induced apoptosis and proliferation inhibition in hepatocarcinoma cells. Cancer Lett.

[40] Uhm JE, Park JO, Lee J (2009). A phase II study of oxaliplatin in combination with doxorubicin as first-line systemic chemotherapy in patients with inoperable hepatocellular carcinoma. Cancer Chemother Pharmacol.

[41] Ding ZB, Hui B, Shi YH (2011). Autophagy activation in hepatocellular carcinoma contributes to the tolerance of oxaliplatin via reactive oxygen species modulation. Clin Cancer Res.

[42] Manov I, Pollak Y, Broneshter R, Iancu TC (2011). Inhibition of doxorubicin-induced autophagy in hepatocellular carcinoma Hep3B cells by sorafenib - the role of extracellular signal-regulated kinase counteraction. FEBS J.

[43] Huynh H, Nguyen TT, Chow KH (2003). Over-expression of the mitogen-activated protein kinase (MAPK) kinase (MEK)-MAPK in hepatocellular carcinoma: its role in tumor progression and apoptosis. BMC Gastroenterol.

[44] Wilhelm S, Carter C, Lynch M (2006). Discovery and development of sorafenib: a multikinase inhibitor for treating cancer. Nat Rev Drug Discov.

[45] Zhang X, Yang XR, Huang XW (2012). Sorafenib in treatment of patients with advanced hepatocellular carcinoma: a systematic review. Hepatobiliary Pancreat Dis Int.

[46] Xie B, Wang DH, Spechler SJ (2012). Sorafenib for treatment of hepatocellular carcinoma: a systematic review. Dig Dis Sci.

[47] Shimizu S, Takehara T, Hikita H (2012). Inhibition of autophagy potentiates the antitumor effect of the multikinase inhibitor sorafenib in hepatocellular carcinoma. Int J Cancer.

[48] Shi YH, Ding ZB, Zhou J (2011). Targeting autophagy enhances sorafenib lethality for hepatocellular carcinoma via ER stress-related apoptosis. Autophagy.

[49] Hayashi-Nishino M, Fujita N, Noda T (2009). A subdomain of the endoplasmic reticulum forms a cradle for autophagosome formation. Nat Cell Biol.

[50] Hui B, Shi YH, Ding ZB (2012). Proteasome inhibitor interacts synergistically with autophagy inhibitor to suppress proliferation and induce apoptosis in hepatocellular carcinoma. Cancer.

[51] Yu HC, Hou DR, Liu CY (2013). Cancerous inhibitor of protein phosphatase 2A mediates bortezomib-induced autophagy in hepatocellular carcinoma independent of proteasome. PLoS One.

[52] Guo XL, Li D, Sun K (2013). Inhibition of autophagy enhances anticancer effects of bevacizumab in hepatocarcinoma. J Mol Med (Berl).

[53] Tai WT, Shiau CW, Chen HL (2013). Mcl-1-dependent activation of Beclin 1 mediates autophagic cell death induced by sorafenib and SC-59 in hepatocellular carcinoma cells. Cell Death Dis.

[54] Gao M, Yeh PY, Lu YS (2008). OSU-03012, a novel celecoxib derivative, induces reactive oxygen species-related autophagy in hepatocellular carcinoma. Cancer Res.

[55] Yu HC, Lin CS, Tai WT (2013). Nilotinib induces autophagy in hepatocellular carcinoma through AMPK activation. J Biol Chem.

[56] Soliman H, Ringash J, Jiang H (2013). Phase II trial of palliative radiotherapy for hepatocellular carcinoma and liver metastases. J Clin Oncol.

[57] Anbalagan S, Pires IM, Blick C (2012). Radiosensitization of renal cell carcinoma in vitro through the induction of autophagy. Radiother Oncol.

[58] Cerniglia GJ, Karar J, Tyagi S (2012). Inhibition of autophagy as a strategy to augment radiosensitization by the dual phosphatidylinositol 3-kinase/mammalian target of rapamycin inhibitor NVP-BEZ235. Mol Pharmacol.

[59] Bristol ML, Di X, Beckman MJ (2012). Dual functions of autophagy in the response of breast tumor cells to radiation: cytoprotective autophagy with radiation alone and cytotoxic autophagy in radiosensitization by vitamin D 3. Autophagy.

[60] Wilson EN, Bristol ML, Di X (2011). A switch between cytoprotective and cytotoxic autophagy in the radiosensitization of breast tumor cells by chloroquine and vitamin D. Horm Cancer.

[61] Altmeyer A, Jung AC, Ignat M (2010). Pharmacological enhancement of autophagy induced in a hepatocellular carcinoma cell line by high-LET radiation. Anticancer Res.

[62] Altmeyer A, Ignat M, Denis JM (2011). Cell death after high-LET irradiation in orthotopic human hepatocellular carcinoma in vivo. In Vivo.

[63] Gao L, Song JR, Zhang JW (2013). Chloroquine promotes the anticancer effect of TACE in a rabbit VX2 liver tumor model. Int J Biol Sci.

[64] Ochsner M (1997). Photophysical and photobiological processes in the photodynamic therapy of tumours. J Photochem Photobiol B.

[65] Andrzejak M, Price M, Kessel DH (2011). Apoptotic and autophagic responses to photodynamic therapy in 1c1c7 murine hepatoma cells. Autophagy.

[66] Arum CJ, Anderssen E, Viset T (2010). Cancer immunoediting from immunosurveillance to tumor escape in microvillus-formed niche: a study of syngeneic orthotopic rat bladder cancer model in comparison with human bladder cancer. Neoplasia.

[67] Liang X, De Vera ME, Buchser WJ (2012). Inhibiting systemic autophagy during interleukin 2 immunotherapy promotes long-term tumor regression. Cancer Res.

[68] Li P, Du Q, Cao Z (2012). Interferon-gamma induces autophagy with growth inhibition and cell death in human hepatocellular carcinoma (HCC) cells through interferon-regulatory factor-1 (IRF-1). Cancer Lett.

[69] Su S, Zhou H, Xue M (2013). Anti-tumor efficacy of a hepatocellular carcinoma vaccine based on dendritic cells combined with tumor-derived autophagosomes in murine models. Asian Pac J Cancer Prev.

[70] Leithead JA, Armstrong MJ, Corbett C (2013). Hepatic ischemia reperfusion injury is associated with acute kidney injury following donation after brain death liver transplantation. Transpl Int.

[71] Yasuda H, Leelahavanichkul A, Tsunoda S (2008). Chloroquine and inhibition of Toll-like receptor 9 protect from sepsis-induced acute kidney injury. Am J Physiol Renal Physiol.

[72] Hoshino A, Matoba S, Iwai-Kanai E (2012). p53-TIGAR axis attenuates mitophagy to exacerbate cardiac damage after ischemia. J Mol Cell Cardiol.

[73] Gotoh K, Lu Z, Morita M (2009). Participation of autophagy in the initiation of graft dysfunction after rat liver transplantation. Autophagy.

[74] Degli Esposti D, Sebagh M, Pham P (2011). Ischemic preconditioning induces autophagy and limits necrosis in human recipients of fatty liver grafts, decreasing the incidence of rejection episodes. Cell Death Dis.

[75] Toso C, Merani S, Bigam DL (2010). Sirolimus-based immunosuppression is associated with increased survival after liver transplantation for hepatocellular carcinoma. Hepatology.

[76] Yang ZJ, Chee CE, Huang S, Sinicrope FA (2011). The role of autophagy in cancer: therapeutic implications. Mol Cancer Ther.

[77] Toshima T, Shirabe K, Matsumoto Y (2013). Autophagy enhances hepatocellular carcinoma progression by activation of mitochondrial beta-oxidation. J Gastroenterol.

[78] Gozuacik D, Kimchi A (2004). Autophagy as a cell death and tumor suppressor mechanism. Oncogene.

[79] Kanzawa T, Kondo Y, Ito H (2003). Induction of autophagic cell death in malignant glioma cells by arsenic trioxide. Cancer Res.

[80] Kim EH, Sohn S, Kwon HJ (2007). Sodium selenite induces superoxide-mediated mitochondrial damage and subsequent autophagic cell death in malignant glioma cells. Cancer Res.

[81] Dupere-Richer D, Kinal M, Menasche V (2013). Vorinostat-induced autophagy switches from a death-promoting to a cytoprotective signal to drive acquired resistance. Cell Death Dis.

[82] Takahashi A, Kimura T, Takabatake Y (2012). Autophagy guards against cisplatin-induced acute kidney injury. Am J Pathol.

[83] Ching JK, Weihl CC (2013). Rapamycin-induced autophagy aggravates pathology and weakness in a mouse model of VCP-associated myopathy. Autophagy.

